# The potential roles of exosomes in pathological cardiomyocyte hypertrophy mechanisms and therapy: A review

**DOI:** 10.1097/MD.0000000000037994

**Published:** 2024-04-26

**Authors:** Lijun Zhang, Fang Xie, Fengmei Zhang, Beiyao Lu

**Affiliations:** a Department of Cardiovascular Surgery, West China Hospital, Sichuan University, Chengdu, China.

**Keywords:** biomarkers, exosomes, mechanisms, pathological cardiomyocyte hypertrophy, therapeutic potential

## Abstract

Pathological cardiac hypertrophy, characterized by the enlargement of cardiac muscle cells, leads to serious cardiac conditions and stands as a major global health issue. Exosomes, comprising small lipid bilayer vesicles, are produced by various cell types and found in numerous bodily fluids. They play a pivotal role in intercellular communication by transferring bioactive cargos to recipient cells or activating signaling pathways in target cells. Exosomes from cardiomyocytes, endothelial cells, fibroblasts, and stem cells are key in regulating processes like cardiac hypertrophy, cardiomyocyte survival, apoptosis, fibrosis, and angiogenesis within the context of cardiovascular diseases. This review delves into exosomes’ roles in pathological cardiac hypertrophy, first elucidating their impact on cell communication and signaling pathways. It then advances to discuss how exosomes affect key hypertrophic processes, including metabolism, fibrosis, oxidative stress, and angiogenesis. The review culminates by evaluating the potential of exosomes as biomarkers and their significance in targeted therapeutic strategies, thus emphasizing their critical role in the pathophysiology and management of cardiac hypertrophy.

## 1. Introduction

Heart failure, a major cause of mortality globally, is frequently precipitated by pathological cardiac hypertrophy.^[1]^ This condition often arises from myocardial stress, which can be due to injury, valvular heart disease, or sustained hypertension.^[2]^ Initially, cardiac hypertrophy acts as a compensatory mechanism to reduce wall stress and enhance cardiac output.^[3,4]^ However, the prolonged presence of hypertrophy leads to a decline in cardiac contractility and, over time, progresses to cardiac decompensation and ultimately heart failure.^[5,6]^ Hypertrophic cardiac growth is characterized by an increase in cardiomyocyte size rather than proliferation, as post-mitotic adult cardiomyocytes lack division capabilities.^[7]^ Numerous pathways, including calcineurin/NFAT, MAPK ERK, small GTP-binding proteins (Ras, Rho), and Protein Kinase C (PKC), have been identified to play pivotal roles in the development of cardiac hypertrophy.^[8]^ These pathways are further complemented by transcriptional and cell surface regulation, as well as the influence of miRNAs, orchestrating the complex process of cardiac hypertrophic growth.^[9]^

Exosomes, small vesicles known for intercellular communication, have recently been recognized for their significant role in various mechanisms.^[10,11]^ They transport biologically active molecules, including proteins, RNA, and DNA, influencing the cellular microenvironment and immune responses.^[12]^ These vesicles not only initiate downstream signaling but also convey genetic material to recipient cells, underscoring their importance in genetic and epigenetic communication.^[13]^ In the realm of cardiovascular diseases, exosomes have demonstrated potential as novel therapeutic agents.^[14]^ Their role extends to diagnostic and therapeutic applications, such as serving as biomarkers and mediators in ischemic heart disease, heart failure, and atherosclerosis.^[15–18]^ These exosomes contribute significantly to cardioprotection by carrying and transferring cardioprotective factors to the damaged heart cells, thus regulating intracellular cell signaling, proliferation, differentiation, apoptosis, and stress response.^[19–22]^

Despite considerable progress in exosome research, the full spectrum of their biological functions in pathological cardiomyocyte hypertrophy has yet to be comprehensively clarified. This review delves into the molecular intricacies of cardiomyocyte hypertrophy and its links to exosomes, scrutinizes the current landscape of treatment modalities, and particularly emphasizes the role of exosomes in enhancing precision therapy for this condition.

## 2. The biogenesis and functions of exosomes

**Figure 1.**
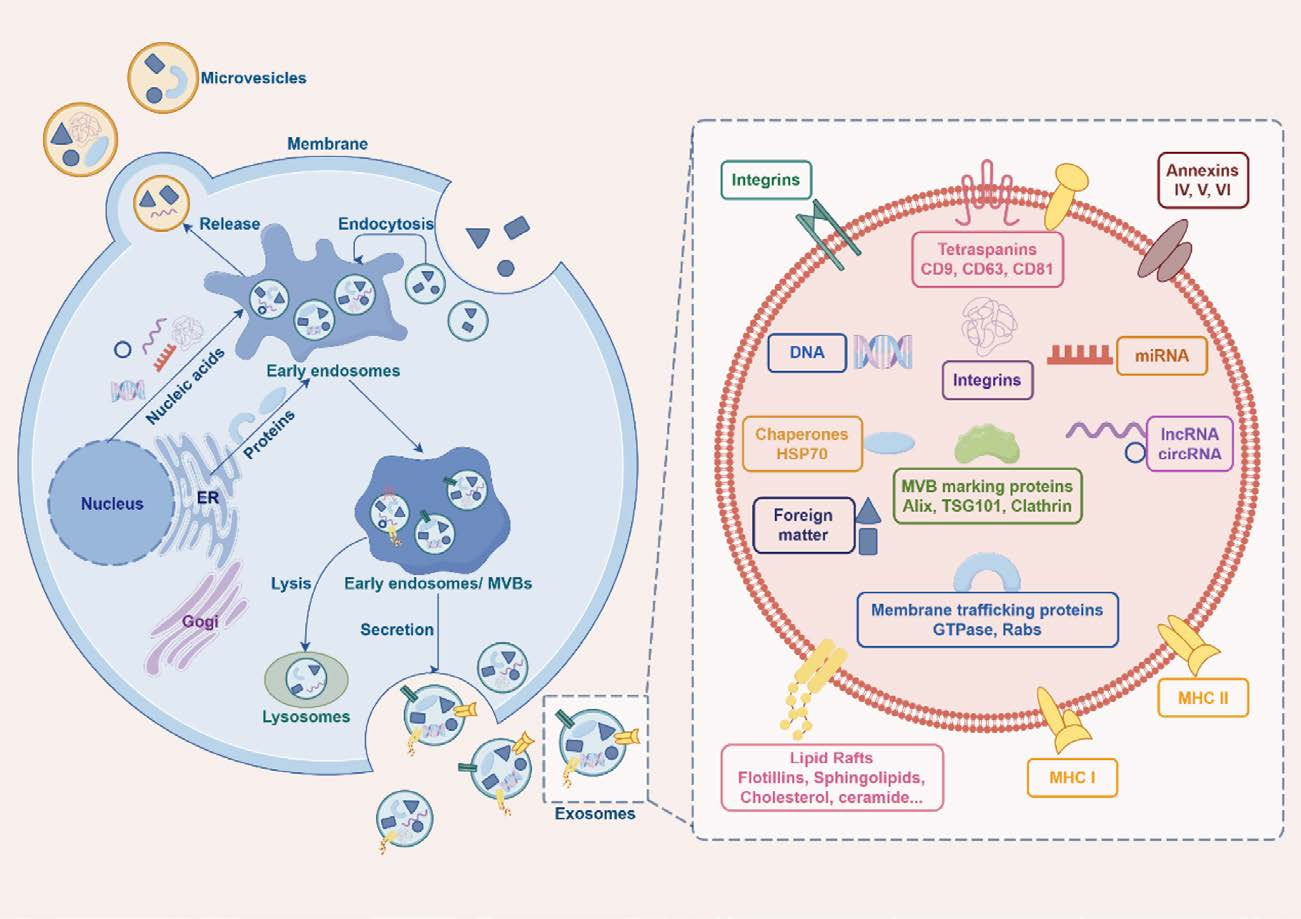
The biogenesis and contents of exosomes.

Exosomes, tiny vesicles approximately 30 to 150 nm in diameter, are released into the extracellular matrix by a variety of cell types.^[23,24]^ These vesicles, formed through endocytosis, encapsulate extracellular proteins, various components, and cell membrane receptors, and are initially known as early endosomes.^[23,25,26]^ The transition of early endosomes into late endosomes, also referred to as multivesicular bodies (MVBs), involves the sorting and accumulation of cargo molecules on the membranes of early endosomes and the creation of intraluminal vesicles through the inward folding of these membranes.^[27]^ The formation of MVBs can proceed via pathways that either depend on or are independent of endosomal sorting complexes required for transport.^[28–30]^ These MVBs have 2 potential fates: they can either merge with lysosomes, leading to their degradation, or fuse with the plasma membrane, releasing intraluminal vesicles externally as exosomes or forming exosomes directly through budding from the cytoplasmic membrane.^[26–28]^ The suppression of exosome secretion is linked to an increase in lysosomal degradation of MVBs.^[29]^ The discharge of exosomes and their integration with target cells are facilitated by the Ras superfamily and Rab proteins (Rab 2B, 5A, 7, 9A, 11, 27, and 35), which are essential for MVB transport.^[28,30]^ Additionally, Ral A/B GTPases enhance exosome secretion by regulating various effector proteins and lipids, such as phospholipase D1, involved in MVB homeostasis, and PLD2, which participates in the budding of exosome cargoes.^[30–32]^ Rab GTPase also aids in the formation of tetrameric coiled-coil complexes at exosomal and receptor cell membranes by recruiting tethering proteins, ensuring the membranes stay in close contact.^[33]^ Exosomes also contain a range of proteins, including members of the transmembrane 4 superfamily (CD63, CD81, and CD9), flotillin, Alix, and TSG101, all of which are involved in the biogenesis of exosomes.^[30]^ The intricate processes of biogenesis, cargo selection, and transfer contribute to the significant heterogeneity observed in exosomes (Fig. 1).

## 3. Exosomes and pathological cardiomyocyte hypertrophy mechanisms

**Figure 2.**
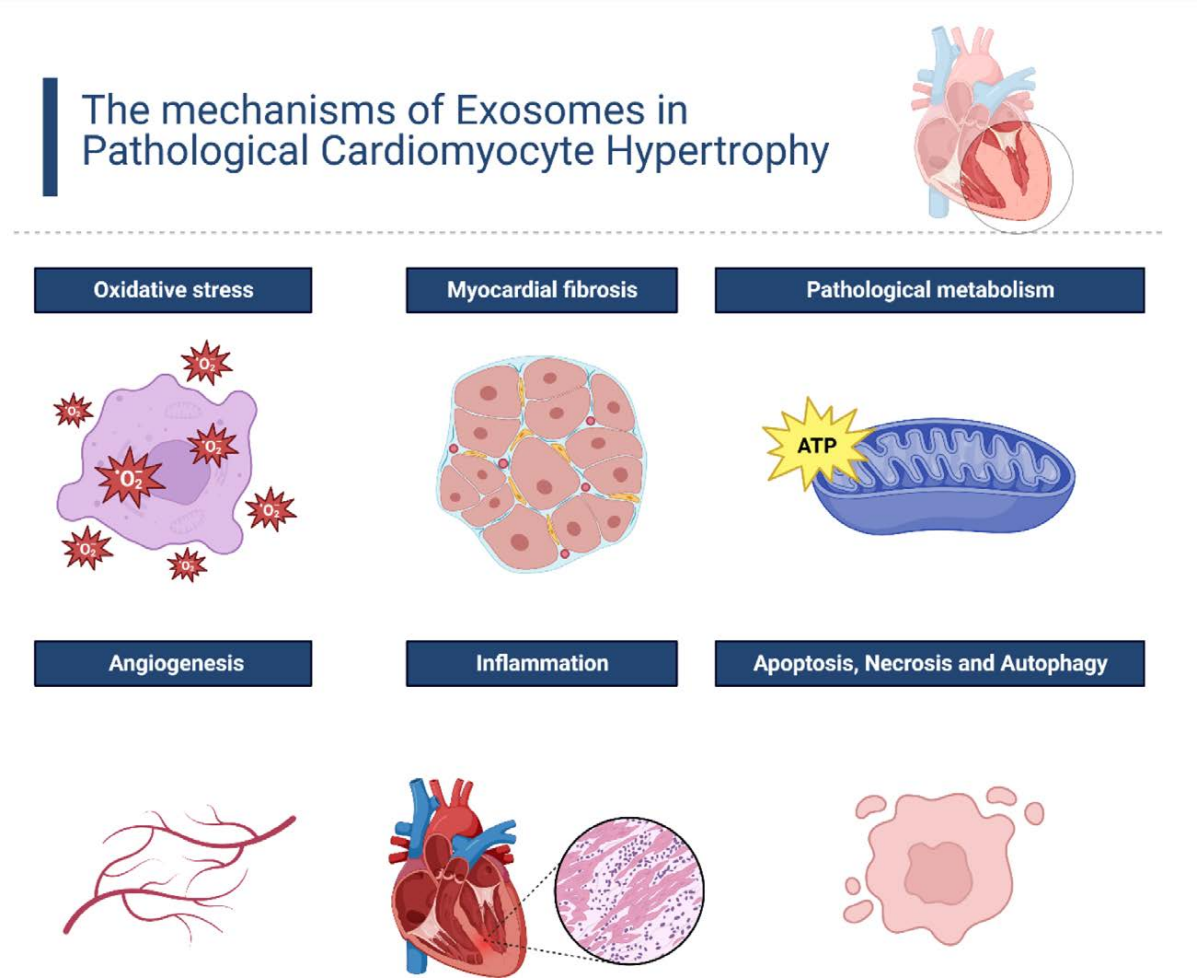
The multifaceted roles of exosomes in the development of pathological cardiomyocyte hypertrophy.

The heart, composed of multiple cell types including cardiomyocytes (30% of total cells but 70 to 80% of the heart’s mass), fibroblasts, and others, undergoes hypertrophy largely through cardiomyocyte enlargement.^[34–36]^ Cardiac hypertrophy involves extensive intracellular changes such as pathological metabolism, myocardial fibrosis, oxidative stress, cell death including necrosis, apoptosis, and autophagy, inflammation, and gene alterations. Additionally, it includes extracellular matrix changes like fibrosis and angiogenesis. Exosomes are significantly implicated in these processes, influencing the wide range of alterations associated with cardiac hypertrophy (Fig. 2).

### 3.1. Pathological metabolism

In pathological cardiac hypertrophy, the heart undergoes a critical metabolic shift from fatty acid oxidation to increased glucose utilization, known as a “substrate switch,” due to altered expression of transcriptional regulators like PPARα, PGC1-α, and HIF1-α.^[37–41]^ This shift, while enhancing glycolytic rates, fails to proportionately increase glucose oxidation, leading to a mismatch that limits acetyl-coA for the TCA cycle and reduces ATP production.^[42,43]^ The hypertrophied heart, facing increased workload and hypoxia, relies more on glucose metabolism, akin to fetal cardiac metabolism, to meet its oxygen-efficient ATP needs.^[34,44]^ However, this adaptation is insufficient to sustain energy demands, as evidenced by depleted phosphocreatine (PCr)/ATP ratios, indicative of a strained creatine kinase energy shuttle system.^[45,46]^ This metabolic dysfunction, characterized by diminished PCr and ATP levels and impaired energy delivery to myofibrils, contributes to contractile dysfunction and progresses to heart failure, with the PCr/ATP ratio emerging as a more accurate predictor of mortality than ejection fraction.^[47,48]^

Mesenchymal stromal cell (MSC)-derived exosomes have emerged as crucial modulators in the metabolic reprogramming associated with pathological cardiac hypertrophy. In models of ischemia followed by reperfusion, the administration of purified MSC-derived exosomes prior to reperfusion has demonstrated the restoration of ATP and NADH levels in myocardial tissue, concurrently reducing oxidative stress. This pivotal finding indicates that MSC-derived exosomes, equipped with a complete set of glycolytic enzymes, facilitate the reestablishment of glycolysis in the reperfused myocardium.^[19]^ Such a role not only provides novel insights into understanding and treating the metabolic imbalances in cardiac hypertrophy, but also suggests that exosomes may enhance glycolytic processes to meet the altered metabolic demands in pathological cardiac hypertrophy.^[49]^

### 3.2. Myocardial fibrosis

Fibrosis in the heart, characterized by the excessive accumulation of extracellular matrix (ECM) proteins like collagens, fibronectin, matrix metalloproteinases (MMPs), and their inhibitors (TIMPs), is a hallmark of various pathological cardiac conditions.^[50]^ Normally, cardiac fibroblasts within the ECM produce and regulate components such as collagen type I and III, maintaining a balance between collagen synthesis and degradation.^[51]^ This process is crucial for providing structural support to cardiomyocytes and facilitating their mechanical, chemical, and electrical functions.^[52]^ However, in response to cardiac injury (e.g., myocardial infarction) or chronic stressors like pressure or volume overload, pro-fibrotic pathways are activated.^[50]^ This involves not just fibroblasts but also other cells like macrophages and lymphocytes, which contribute to fibrosis either directly or through the secretion of fibrogenic mediators like TNF-α, TGF-β, and endothelin-1.^[50]^ A key event in this process is the transformation of fibroblasts into myofibroblasts, which have increased proliferative and secretory capabilities and migrate to injury sites, aiding in tissue repair.^[52]^ However, chronic activation of these cells leads to abnormal collagen deposition and accumulation, resulting in mechanical stiffening of the heart. This stiffening impairs both diastolic and systolic function and increases the risk of arrhythmias by disrupting electrical conduction within the heart.^[53]^

Recent studies have demonstrated the significant role of exosomes in cardiac fibrosis. For instance, Yang et al (2018) discovered that exosomes derived from cardiomyocytes, enriched with miR-208a, play a critical role in fibroblast activation.^[54]^ At the molecular level, miR-208a is shown to enhance NFAT phosphorylation by targeting Dyrk2, preventing its entry into the nucleus in cardiac fibroblasts (CFs), thereby triggering fibrosis.^[54]^ This indicates a direct involvement of cardiomyocyte-derived exosomes in promoting fibrotic responses. Additionally, Chaturvedi et al (2015) found that during exercise, exosomes enriched with miRNA-29b and miRNA-455 from cardiomyocytes can actually prevent fibrosis.^[55]^ This prevention is achieved by downregulating MMP9 levels in diabetic mice, demonstrating the potential of specific exosomal miRNAs in mitigating fibrosis.^[55]^ These studies highlight the dual nature of exosome involvement in cardiac fibrosis, both in its promotion and prevention, depending on the specific miRNAs they carry.

### 3.3. Oxidative stress

Oxidative stress in the heart arises from an imbalance between the production of reactive oxygen species (ROS) and the heart’s ability to neutralize them using its antioxidant systems, such as superoxide dismutase, catalase, and glutathione peroxidase.^[56]^ Excessive ROS production is linked to pathological cardiac hypertrophy and heart failure (HF) in both humans and animal models.^[57–60]^ The heart primarily generates ROS through 3 sources: the NADPH oxidase enzyme complex, the mitochondrial respiratory chain, and uncoupled endothelial nitric oxide synthase (eNOS).^[57]^ The accumulation of ROS from these sources is associated with various cardiac diseases, and controlling ROS levels either genetically or pharmacologically has been shown to influence cardiac remodeling negatively. Hypertrophic stimuli like angiotensin II (Ang II), endothelin-1, catecholamines, cytokines, and biomechanical stretch can increase ROS production in cardiomyocytes.^[61,62]^ This increase in ROS activates hypertrophic signaling mediators and transcription factors such as ERK1/2 and NF-κB, leading to pathological changes.^[63]^ Specifically, ROS produced by NADPH oxidase or mitochondria in pathological cardiac conditions contribute to hypertrophy, fibrosis, reduced contractility, and apoptosis.^[60,63–65]^ This indicates that managing oxidative stress is crucial for preventing adverse cardiac remodeling and associated pathologies.

Recent studies have revealed that exosomes carry proteins and microRNAs (miRNAs) which directly influence cellular responses to oxidative stress. For instance, exosomes derived from cardiac progenitor cells under oxidative stress conditions are loaded with upregulated miR-21.^[66]^ These exosomes enter target cells through membrane fusion, inhibiting PDCD4 and cleaved caspase-3 in the target cells.^[66]^ This action effectively reduces cardiomyocyte apoptosis. Additionally, during ischemia and hypoxia, exosomes mediate the biochemical and cellular activities in the ischemic area, thereby correcting the cascade reaction induced by ischemia and hypoxia, and preventing the onset of pathological conditions related to oxidative stress.^[67]^ These findings demonstrate the therapeutic potential of exosomes in attenuating oxidative stress-induced cardiac damage, highlighting their importance in cardiac repair and the treatment of heart diseases.

### 3.4. Apoptosis, necrosis and autophagy

In the development of cardiac hypertrophy, the interplay of apoptosis, necrosis, and autophagy is crucial. Under normal conditions, apoptosis occurs at a very low rate in the heart, but in the setting of heart disease, including cardiac hypertrophy, the rate of cardiomyocyte apoptosis increases significantly.^[36]^ This increased apoptosis, marked by cell shrinkage and formation of apoptotic bodies, contributes to the progression from compensated to decompensated heart growth and eventual heart failure.^[68]^ Necrosis, in contrast to the controlled process of apoptosis, is characterized by loss of membrane integrity and swelling of cells and organelles, often leading to an inflammatory response.^[69]^ This uncontrolled cell death mechanism exacerbates the pathological remodeling seen in hypertrophic hearts. Autophagy, a cellular process for degrading and recycling components, usually protects cardiomyocytes by clearing damaged proteins and organelles.^[34]^ Under cardiac stress, as seen in hypertrophy, autophagy levels increase to manage the increased synthesis of proteins and aid in sarcomeric remodeling.^[70]^ However, excessive autophagy, particularly in the context of persistent cardiac stress, can lead to cell death, contributing to cardiac dysfunction.^[71]^ The balance among apoptosis, necrosis, and autophagy is thus a delicate one, where a shift towards any one can significantly impact the progression of cardiac hypertrophy. While each of these processes can independently contribute to cardiac pathology, their interaction and the context of the stressors involved play a critical role in determining the heart’s response to hypertrophic stimuli.

Exosomes derived from induced pluripotent stem cell-derived cardiomyocytes (iCM-Ex) have been shown to play a significant role in modulating autophagy in cardiomyocytes.^[72]^ These exosomes are effective in upregulating autophagy, thereby maintaining cardiac homeostasis.^[72]^ ICM-Ex modulates signaling pathways upstream of autophagy, suggesting their involvement in the initial steps of the autophagic process.^[72]^ Furthermore, iCM-Ex has been demonstrated to restore impaired autophagic flux in ischemic conditions, highlighting their potential in reversing autophagy-related dysfunctions in cardiac hypertrophy.^[72]^

### 3.5. Inflammation

Pathological events like pressure overload or myocardial infarction activate the innate immune system, leading to inflammation in the heart.^[73,74]^ This is marked by increased levels of pro-inflammatory cytokines such as TNF-α, TLR, and interleukins.^[75,76]^ While initial inflammation can be adaptive and aid in cardiac repair, chronic inflammation contributes to impaired heart function, increased ROS production, apoptosis, fibrosis, and eventually leads to maladaptive cardiac remodeling and heart failure.^[77,78]^ A recent study highlights that miR-155-enriched exosomes from hypertrophic cardiomyocytes activate macrophages via the MAPK pathway, increasing inflammatory cytokine production and exacerbating cardiac hypertrophy.^[79]^ This finding emphasizes the significant role of these exosomes in driving cardiac inflammation.

### 3.6. Angiogenesis

Angiogenic processes are integral to cardiac remodeling, orchestrated through paracrine communication between cardiomyocytes and vascular cells.^[80,81]^ Angiogenesis in cardiac tissue is considered crucial for preserving blood supply and nutrients to enlarging heart muscle cells. Impairment of this angiogenic response during the adaptive enlargement of the heart can result in diminished muscle contraction.^[82,83]^ Conversely, angiogenic facilitation under conditions of pressure overload serves a cardioprotective role, staving off the progression from adaptive cardiac hypertrophy to heart failure.^[84]^ Empirical evidence from beneficial physiological hypertrophy models indicates a preserved or augmented myocardial capillary network, correlating positively with the left ventricular mass index in patients with aortic stenosis who exhibit conserved ejection fraction, indicative of compensatory hypertrophy.^[85–87]^ Contrastingly, the transition towards advanced pathological remodeling and heart failure is characterized by a marked diminution in myocardial capillary density, suggesting that therapeutic strategies aimed at modulating angiogenesis may hold promise in managing cardiac hypertrophy and its sequelae.^[88,89]^ Recent studies reveal that exosomal MiR-29a, released from cardiomyocytes, engages in direct interaction with cardiac microvascular endothelial cells.^[90]^ This interaction involves MiR-29a molecules targeting and subsequently downregulating VEGFA expression in endothelial cells, thereby significantly altering their functional properties.^[90]^ This alteration impacts critical endothelial cell processes, including proliferation, migration, and tube formation.^[90]^ As a result, exosomes play a pivotal role as mediators in the angiogenic response within the cardiac microenvironment, exerting a substantial influence on the processes of cardiac hypertrophy and remodeling.^[90]^

## 4. Exosomes and signaling pathways in pathological cardiac hypertrophy

Pathological cardiac hypertrophy involves complex signaling pathways influenced by exosomes, which are integral to the communication between cardiomyocytes and other cardiac cells. This section aims to explore how exosomes affect these signaling pathways, particularly in the context of the transition from compensated heart growth to decompensated growth leading to heart failure. We will highlight the role of exosomal content in modulating these signaling cascades, thereby impacting the progression of pathological cardiac hypertrophy. Identifying exosome-mediated pathways offers a promising avenue for potential therapeutic interventions.

### 4.1. GPCR pathways

G Protein-Coupled Receptors (GPCRs) are activated by factors elevated during cardiac stress and HF, with Gαq, a subunit of G proteins, playing a key role in pathological cardiac hypertrophy. GPCRs like the Ang II receptor type 1, endothelin receptors, and α1-adrenergic receptors are activated by hormones like Ang II, leading to downstream signaling involving proteins such as phospholipase C and MAPKs.^[34]^ Studies show that manipulating GPCR signaling can influence heart growth, with overexpression of Gαq causing HF and reduced signaling leading to less hypertrophy.^[91–93]^ Current therapies targeting GPCR pathways include ACE inhibitors and β-blockers. Adrenergic receptors (Ars), particularly α1 and β subtypes, have distinct roles in cardiac function and hypertrophy, influencing the transition to HF.^[94,95]^ A study explores the role of exosomes in modulating ciliary signaling of GPCRs.^[96]^ It reveals that when GPCRs cannot be retrieved back into the cell, they accumulate at the ciliary tips and are released into exosomes, facilitated by an actin network involving actin regulators like Drebrin and Myosin 6.^[96]^ This novel mechanism suggests a critical role for exosomes in GPCR signaling within cilia, potentially impacting cellular communication and disease processes.^[96]^

### 4.2. PI3K (p110γ) signaling

PI3K (p110γ), activated by GPCR pathways, differs from PI3K (p110α) in its influence on cardiomyocyte contractility, primarily through the modulation of phosphodiesterases and cAMP.^[97]^ This enzyme’s activity is heightened in response to cardiac stress, with its role in diseased hearts varying by stress type. Transgenic models with depleted PI3K (p110γ) exhibit higher basal contractility, yet increased susceptibility to pressure overload and ischemic injury, but are resistant to isoproterenol-induced heart failure.^[97–101]^ Kinase-inactive or overexpressed inactive forms of PI3K (p110γ) show less hypertrophy and fibrosis under stress,^[102]^ and are protected from ischemia-reperfusion injury.^[103]^ Prolonged inactivation of both PI3K (p110α) and (p110γ) leads to pathological remodeling.^[104]^ While PI3K (p110γ), activated by GPCR pathways, primarily modulates cardiomyocyte contractility through phosphodiesterases and cAMP, recent research bridges this pathway with the role of exosomes.^[105]^ HUVEC-derived exosomes, in acute myocardial infarction, activate the PI3K/AKT signaling in cardiomyocytes, enhancing cardiac function and reducing fibrosis.^[105]^ This connection suggests that exosomes could influence cardiomyocyte response to stress, possibly through the PI3K/AKT pathway, offering a novel perspective on the interplay between exosomal mechanisms and cardiac stress responses.

### 4.3. PKCα and PKCβ

PKCα and PKCβ isoforms are closely associated with maladaptive heart conditions. Elevated levels of PKCα and PKCβ have been observed in failing human hearts.^[106]^ In genetic mouse models, PKCα has been identified as a contributor to contractile dysfunction; mice overexpressing PKCα exhibited diminished contractile function, while deletion of PKCα resulted in enhanced cardiac contractility.^[107–109]^ Similarly, increased activity of PKCβ leads to pathological heart growth. Transgenic mice with cardiac-specific overexpression of PKCβ displayed heart enlargement, dysfunction, fibrosis, and early mortality.^[110–112]^ Although not essential, as PKCβ-null mice showed similar hypertrophic responses to stress as wild-type mice,^[113]^ inhibition of PKCβ, as shown in a rat model of diabetic cardiomyopathy, reduced myocyte hypertrophy and fibrosis.^[114]^

Current literature does not clearly define the direct relationship between exosomes and PKCα and PKCβ in cardiac hypertrophy. However, studies like those by Herranz et al and Gosselin et al suggest a close link between Protein Kinase C (PKC) and exosome secretion.^[115,116]^ Herranz et al explored PKCδ’s role in regulating T cell exosome release, emphasizing its impact on actin reorganization at the immunological synapse, crucial for exosome release.^[115]^ Gosselin et al discussed how PKC activation in astrocytes affects excitatory amino-acid transporters’ distribution into exosomes, indicating PKC’s influence on exosome-mediated neural functions.^[116]^ These insights pave the way for future research on PKC’s role in modulating exosomes in cardiac hypertrophy.

### 4.4. Calcineurin and CaMK

Calcineurin and Ca2+/calmodulin-dependent protein kinase II (CaMKII), both calcium-dependent proteins, are implicated in cardiac hypertrophy and remodeling. Calcineurin activates nuclear factor of activated T-cells (NFAT), promoting pro-hypertrophic gene transcription, and its elevated activity correlates with HF and hypertrophy.^[117,118]^ Transgenic mice studies confirm calcineurin’s role in pathological hypertrophy and HF, with its inhibition reducing hypertrophy.^[119,120]^ CaMKII, activated by Gq signaling and oxidative stress, induces hypertrophy and HF, as evidenced in CaMKIIδc overexpression and knockout mouse models.^[93,121,122]^ Recent studies also reveal interactions between calcineurin–NFAT and CaMKII in cardiac response to stress and remodeling.^[123,124]^ The study on miR-214-enriched exosomes from BMSCs complements the understanding of cardiac hypertrophy and remodeling.^[125]^ These exosomes, targeting CaMKII, offer a novel approach to mitigating oxidative stress-induced damage in cardiac cells.^[125]^ This mechanism parallels the known roles of calcineurin and CaMKII in cardiac hypertrophy, suggesting a potential therapeutic synergy.^[125]^ Understanding how these exosomes influence calcium-dependent pathways could provide insights into new strategies for treating heart failure and hypertrophy.

### 4.5. Histone deacetylases

Histone deacetylases (HDACs) play a pivotal role in regulating gene expression in pathological cardiac hypertrophy, primarily through chromatin remodeling that affects gene transcription.^[126,127]^ Within the HDAC superfamily, divided into 4 classes, Classes I, II, and IV are zinc-dependent, whereas Class III operates via an NAD-dependent mechanism.^[128–131]^ Class Iia HDACs, despite their lower enzymatic activity compared to other classes, significantly influence cardiac hypertrophy. This influence is mediated through interactions with transcription factors, notably the MEF2 family, and the recruitment of other co-repressors, thereby regulating gene expression.^[129,132–134]^ In this context, Class Iia HDACs serve as key regulators in cardiac hypertrophy. Their genetic deletion in mice models has shown an enhanced hypertrophic response, underscoring their protective role against cardiac stress.^[135,136]^ Interestingly, the hypertrophic response requires the nuclear export of these HDACs, highlighting the complexity of their regulatory mechanisms.^[136,137]^ Furthermore, post-translational modifications, notably phosphorylation, play a crucial role in modulating the activity of Class Iia HDACs. Phosphorylation by enzymes such as CaMKII and PKD leads to their nuclear exclusion and alters their interaction with transcription factors, thus dynamically regulating gene expression in response to cardiac stress.^[138–141]^ This multifaceted regulation of HDACs, particularly Class Iia, underscores their integral role in the cardiac hypertrophic response and presents potential targets for therapeutic interventions.

While existing research has not directly associated exosomes with HDACs in the context of cardiac hypertrophy, there is evidence of their interplay in other diseases. In acute myeloid leukemia, exosomal miR-34a targets histone deacetylase 2 (HDAC2), affecting leukemia stem cell survival and exosome shedding, illustrating potential therapeutic avenues.^[142]^ Similarly, in gastric cancer, exosomal LINC00355 modulates HDAC3 activity, inhibiting TP53INP1 transcription and enhancing epithelial-mesenchymal transition and cancer progression.^[143]^ These studies underscore the intricate dynamics between exosomes and epigenetic regulators in pathological states, suggesting avenues for future cardiac hypertrophy research.

## 5. Exosomes as biomarkers in pathological cardiomyocyte hypertrophy

Exosomes, a subtype of extracellular vesicles, have emerged as promising biomarkers for various pathological conditions, including cardiomyocyte hypertrophy. These nano-sized vesicles are secreted by various cell types in the heart and contain a unique cargo of proteins, lipids, and nucleic acids that reflect the physiological state of the cell.^[144,145]^ In the context of pathological cardiac hypertrophy, exosomes derived from different cardiac cells have been shown to contain specific miRNAs and proteins that could serve as potential biomarkers for diagnosis and prognosis (Table 1).

**Table 1:** Exosomal biomarkers in cardiomyocyte hypertrophy.

Exosome source	Exosomal cargo	Effect	Ref
Cardiomyocytes	miR-181a, miR-217, miR-378	Potential biomarkers for early detection and monitoring	^[146^^–^^148]^
	Hsp20	Lower levels in exosomes from diabetic cardiomyocytes; restoration attenuates cardiac dysfunction, hypertrophy, and fibrosis	^[149,150]^
	Hsp90	Implicated in activation of STAT-3 signaling during cardiac hypertrophy	^[151,152]^
	AT1R	Potential biomarker for pressure overload-induced hypertrophy	^[153]^
Cardiac fibroblasts	miR-146a	Induces cardiac dysfunction in maladaptive hypertrophy by downregulating SUMO1 and modulating Ca2 + cycling	^[154,155]^
	miR-27a	Implicated in progression of cardiac hypertrophy through targeting PDLIM5	^[156^^–^^158]^
	miR-21_3p	Induces cardiomyocyte hypertrophy by silencing SORBS2 and PDLIM5	^[159]^
	miR-133a, miR-208, miR-499	Influence cardiac hypertrophy by regulating genes encoding calcium channels like InsP3R	^[159]^
Myofibroblasts	miR-200a-3p	Impairs endothelial cell functions; mediates endothelial dysfunction	^[160]^
Macrophages	miR-155	Promotes cardiac hypertrophy	^[79,161,162]^
Adipocytes	miR-200a	Decreases TSC1 and activates mTOR, promoting cardiomyocyte hypertrophy	^[163]^
Cardiac fibroblast-induced pluripotent stem cells	miR-22	May alter expression of miR-22, a key regulator of cardiac hypertrophy and remodeling	^[164]^
Cardiac progenitor cells	miR-133a	Protects hearts from pathological hypertrophy, fibrosis, and apoptosis	^[165]^
Cardiosphere-derived cells	Y RNA fragment EV-YF1	Attenuates Ang II-induced cardiac hypertrophy; associated with changes in IL-10 expression	^[166,167]^
Spontaneously hypertensive rats (serum)	AGT, renin, ACE	Contributes to increased Ang II secretion in cardiomyocytes, leading to hypertrophic responses	^[168]^

ACE = angiotensin-converting enzyme, AGT = angiotensinogen, AT1R = angiotensin II type 1 receptor, InsP3R = inositol 1,4,5-trisphosphate receptor, mTOR = mammalian target of rapamycin, PDLIM5 = PDZ and LIM domain 5, SORBS2 = sorbin and SH3 domain-containing protein 2, SUMO1 = small ubiquitin-related modifier 1, TSC1 = tuberous sclerosis complex 1.

### 5.1. Exosomes derived from cardiomyocyte

Cardiomyocytes, the primary cell type in the heart, play a crucial role in the development and progression of pathological cardiac hypertrophy. Several studies have identified miRNAs in cardiomyocyte-derived exosomes that are associated with pathological hypertrophy. For instance, miR-181a, miR-217, and miR-378 have been found to be secreted from cardiomyocytes in response to hypertrophic stimuli.^[146–148]^ The levels of these miRNAs in circulating exosomes could potentially be used as noninvasive biomarkers for the early detection and monitoring of pathological cardiac hypertrophy. Moreover, the expression of miR-181a in exosomes has been shown to be downregulated after treatment with Sacubitril/valsartan, indicating its potential as a biomarker for treatment response.^[146]^

In addition to miRNAs, specific proteins in cardiomyocyte-derived exosomes have also been proposed as biomarkers for pathological hypertrophy. For example, diabetic cardiomyocytes secrete exosomes with lower levels of Hsp20, and the restoration of Hsp20 in these exosomes attenuates cardiac dysfunction, hypertrophy, and fibrosis.^[149,150]^ This suggests that Hsp20 levels in circulating exosomes could serve as a biomarker for diabetes-induced cardiac hypertrophy. Similarly, Hsp90 and IL-6 in cardiomyocyte-secreted exosomes have been implicated in the activation of STAT-3 signaling during cardiac hypertrophy, indicating their potential as biomarkers for this pathological process.^[151,152]^ Furthermore, the presence of AT1R-enriched exosomes released from cardiomyocytes under conditions of transverse aortic constriction suggests that AT1R levels in circulating exosomes could be a biomarker for pressure overload-induced cardiac hypertrophy.^[153]^

### 5.2. Exosomes derived from cardiac fibroblast

Cardiac fibroblasts, the second most abundant cell type in the heart, play a crucial role in the development and progression of pathological cardiac hypertrophy.^[169–171]^ During cardiac stress, fibroblasts proliferate, differentiate into myofibroblasts, and secrete extracellular matrix proteins and proinflammatory cytokines, contributing to cardiac fibrosis and remodeling.^[172]^ Interestingly, studies have shown that the subtype switching of fibroblasts is closely associated with cardiomyocyte hypertrophic responses in the early stages of cardiac hypertrophy.^[170]^ Moreover, co-culture experiments and treatments with fibroblast-conditioned media have demonstrated that factors secreted by fibroblasts can directly influence the hypertrophic response of cardiomyocytes.^[173,174]^

Emerging evidence suggests that exosomes derived from cardiac fibroblasts may serve as potential biomarkers for pathological cardiac hypertrophy. These exosomes contain a variety of miRNAs that can modulate the hypertrophic response in cardiomyocytes. For instance, miR-146a, a negative regulator of immune responses, has been found to induce cardiac dysfunction in maladaptive hypertrophy by downregulating SUMO1 expression and modulating Ca2 + cycling.^[154,155]^ This miRNA is secreted by trans-differentiated fibroblasts and transferred to cardiomyocytes via exosomes in the failing heart. Similarly, miR-27a, another fibroblast-derived miRNA, has been implicated in the progression of cardiac hypertrophy through its targeting of PDZ and LIM domain 5 (PDLIM5).^[156–158]^ Additionally, fibroblast exosomal-derived miR-21_3p (miR-21*) has been identified as a potent paracrine-acting RNA molecule inducing cardiomyocyte hypertrophy by silencing SORBS2 and PDLIM5.^[159]^

Other fibroblast-secreted miRNAs, such as miR-133a, miR-208, and miR-499, have also been shown to influence cardiac hypertrophy by regulating the expression of genes encoding calcium channels, such as the inositol trisphosphate 3 receptor (InsP3R).^[159]^ Furthermore, fibroblast-derived exosomes have been demonstrated to increase the expression of renin-angiotensin system components and enhance angiotensin II production in cardiomyocytes via the activation of mitogen-activated protein kinases (MAPKs) and Akt signaling pathways.^[175]^ Moreover, exosomes derived from TGF-β1-activated myofibroblasts have been shown to impair endothelial cell functions, including angiogenic potential, cell migration, and viability.^[160]^ These exosomes were found to contain altered levels of microRNAs, particularly miR-200a-3p, which played a pivotal role in mediating endothelial dysfunction.^[160]^ This finding suggests that myofibroblast-derived exosomes enriched with miR-200a-3p could serve as biomarkers or therapeutic targets in cardiovascular diseases, highlighting their significant impact on endothelial cell biology.

### 5.3. Exosomes derived from endothelial cells

Endothelial cells play a crucial role in maintaining cardiovascular homeostasis, and their dysfunction has been implicated in the development of various cardiovascular diseases, including pathological cardiac hypertrophy. Endothelial progenitor cells (EPCs)-derived exosomes can modulate endothelial cell survival and proliferation.^[176–178]^ A clinical study showed that hypertensive patients with left ventricular hypertrophy have decreased circulating EPC numbers and adhesive function compared to those without left ventricular hypertrophy.^[179]^ These findings may explain the pathogenetic processes linking left ventricular hypertrophy and endothelial injury in cardiovascular disease.^[180,181]^ Furthermore, EPC-derived exosomes could protect cardiomyocytes from angiotensin II-induced hypertrophy and apoptosis by upregulating Akt/p-Akt and its downstream eNOS/p-eNOS.^[182]^ These studies highlight the potential of endothelial cell-derived exosomes in regulating cardiac hypertrophy and their crosstalk with cardiomyocytes.

### 5.4. Exosomes derived from immune cells in the heart

In addition to endothelial cells, immune cells, particularly macrophages, also contribute to the development of pathological cardiac hypertrophy. Macrophages play a stage-dependent role in the progression of pathological cardiac hypertrophy.^[170]^ Macrophage-derived exosomes containing miR-155 can promote cardiac hypertrophy through various signaling pathways, such as Jaird2 in cardiomyocytes,^[161]^ FoxO3a in uremic cardiomyopathy model,^[162]^ and MAPK pathway in angiotensin II-induced hypertrophic cardiomyocytes.^[79]^ Additionally, macrophage-derived miR-155 can suppress fibroblast proliferation and increase inflammation, leading to impaired cardiac repair after myocardial infarction.^[183]^ The communication between macrophages, fibroblasts, and cardiomyocytes via exosomes shows complex regulatory networks during pathological cardiac hypertrophy, emphasizing the importance of immune cell-derived exosomes in this process.

### 5.5. Exosomes derived from noncardiac cells

Apart from cells within the heart, noncardiac cells, such as adipocytes, can also influence the development of cardiac hypertrophy through exosome-mediated communication. Adipose tissue, as a vital endocrine organ, can modulate myocardial hypertrophy. Activation of PPARγ signaling in adipocytes increases miR-200a expression and secretion via exosomes. Delivery of miR-200a in adipocyte-derived exosomes to cardiomyocytes contributes to decreased TSC1 and subsequent mTOR activation, promoting cardiomyocyte hypertrophy.^[163]^ These results provide insights into the crosstalk between adipocytes and cardiomyocytes in regulating cardiac hypertrophy and remodeling, highlighting the role of noncardiac cell-derived exosomes in this process.

### 5.6. Exosomes derived from stem cells

Stem cell-derived exosomes have emerged as a promising therapeutic approach for pathological cardiac hypertrophy due to their regenerative and cardioprotective properties. Mesenchymal stem cell (MSC)-derived exosomes can protect the myocardium against pathological remodeling and preserve heart function in pressure overload-induced cardiac hypertrophy.^[184]^ Adipose-derived stem cell (ADSC)-derived exosomes can inhibit cardiomyocyte apoptosis and hypertrophy by reducing the expression of PUMA and ETS-1 and regulate anti-inflammatory factors.^[185,186]^ Exosomes secreted from cardiac fibroblast-induced pluripotent stem cells (CF-iPSCs) may alter the expression of miR-22, a key regulator of cardiac hypertrophy and remodeling.^[164]^ Cardiac progenitor cell-derived exosomal miR-133a could protect the hearts from pathological cardiac hypertrophy, fibrosis, and apoptosis.^[165]^ These findings underscore the therapeutic potential of stem cell-derived exosomes in attenuating pathological cardiac hypertrophy and promoting cardiac repair.

### 5.7. Exosomes secreted by cardiosphere-derived cells

Cardiosphere-derived cells (CDCs), a specific type of cardiac progenitor cells, have shown remarkable potential in stimulating cardiac repair and attenuating adverse remodeling.^[187,188]^ CDC-derived exosomes can decrease acute ischemia-reperfusion injury and halt chronic post-MI adverse remodeling in pigs,^[189]^ as well as attenuate pressure overload-induced right ventricular dysfunction in juvenile Yorkshire pigs.^[166]^ Infusions of CDC-derived exosomes or the Y RNA fragment EV-YF1, a CDC-derived noncoding RNA, can attenuate angiotensin II-induced cardiac hypertrophy in association with changes in the expression of the anti-inflammatory cytokine IL-10.^[166,167]^ These studies highlight the therapeutic potential of CDC-derived exosomes in mitigating pathological cardiac hypertrophy and promoting cardiac repair, further emphasizing the importance of stem cell-derived exosomes in this context.

### 5.8. Exosomes derived from animal models of cardiac hypertrophy

Animal models of cardiac hypertrophy, such as spontaneously hypertensive rats (SHR), provide valuable insights into the mechanisms underlying the development and progression of pathological cardiac hypertrophy. Jingwei Yu and colleagues found that SHR serum exosomes (SHR Exo) contain elevated levels of renin-angiotensin system (RAS) proteins, including AGT, renin, and ACE, which contribute to the increased autocrine secretion of angiotensin II (Ang II) in cardiomyocytes.^[168]^ The injection of SHR Exo into mice induced left ventricular wall thickening and decreased cardiac function, while the AT1-type receptor antagonist telmisartan prevented hypertrophy of H9c2 cells induced by SHR Exo. These findings suggest that SHR-derived exosomes play a crucial role in the progression from hypertension to cardiac hypertrophy by carrying RAS proteins into cardiomyocytes, leading to increased Ang II secretion and subsequent hypertrophic responses.

In conclusion, exosomes derived from animal models of cardiac hypertrophy, such as SHR, provide novel insights into the mechanisms underlying the progression from hypertension to cardiac hypertrophy. These exosomes carry specific proteins, such as RAS components, that can induce hypertrophic responses in cardiomyocytes. Targeting these exosomal cargo proteins may represent a promising therapeutic strategy for preventing or treating pathological cardiac hypertrophy.

## 6. Exosome-based treatments for pathological cardiomyocyte hypertrophy

Recent advancements in cardiovascular research have highlighted the significant potential of exosomes in treating cardiac hypertrophy. Ling Gao et al demonstrated that exosomes from human-induced pluripotent stem cell-derived cardiac cells (hiPSC-CCs) effectively treat cardiac hypertrophy.^[190]^ Their combined in vitro and in vivo approaches showed these exosomes improved cardiomyocyte survival and heart function, and lessened scar size, without increasing arrhythmia risks.^[190]^ However, their research did not analyze the specific contents or cargos of the exosomes. In a related study, Romain Gallet and colleagues explored the impact of cardiosphere-derived cell (CDC) exosomes on cardiac hypertrophy.^[189]^ They discovered that Y RNA fragments within these exosomes played a crucial role in reducing scarring and improving cardiac function in porcine models of myocardial infarction, which often leads to cardiac hypertrophy.^[189]^ These Y RNA fragments significantly contributed to decreased infarct size, preserved left ventricular function, and improved cardiac remodeling in scenarios of cardiac hypertrophy.^[189]^ This highlights the potential of CDC-derived exosomes, especially through targeting specific contents like Y RNA fragments, as a promising treatment approach for conditions leading to cardiac hypertrophy (Table 2).^[189]^

**Table 2:** Exosome-associated therapeutic strategies for cardiomyocyte hypertrophy.

Components	Experiment	Functions	Mechanism	Ref
miR-148a	In vitro	Against myocardial hypertrophy	Modulation of Ang II-induced hypertrophic response in NRCMs	^[151]^
miR-181a	In vitro and In vivo	Attenuates myocardial fibrosis and hypertrophy	Downregulates fibrotic genes Fn1, Col1, Vim, and hypertrophic markers Nppa, Nppb	^[152]^
miR-29a	In vitro	Treating cardiac fibrosis.	Targeting TGFβR I	^[153]^
miR-10a	In vitro	Alleviates cardiac hypertrophy in cultured cardiomyocytes	Tnhibite TBX5 protein expression	^[169]^
miR-302b-3p and miR-373-3p	In vitro and In vivo	Promotes the proliferation of cardiomyocytes	Regulates the HIPPO signal transduction pathway	^[170]^
circ_0018553	In vitro	Protects against Ang II-induced cardiac hypertrophy	Inhibits miR-4731, which targets SIRT2, reducing Ang II-induced cardiac hypertrophy.	^[171]^

NRCMs = neonatal rat cardiomyocytes, TBX5 = T-box 5, TGFβR I = transforming growth factor β receptor I.

### 6.1. Treating with exosomal miRNAs

Following these discoveries, extensive research has identified a variety of cargo components within exosomes, particularly microRNAs (miRs), which play a significant role in treating cardiac hypertrophy. Mao et al successfully demonstrated that genetically engineered heart-targeting exosomes (HHP-EXO), derived from human cardiosphere-derived cells (CDCs), are effective in treating cardiac hypertrophy.^[191]^ By engineering HHP-EXO to display a heart homing peptide, they ensured enhanced targeting and internalization in cardiomyocytes.^[191]^ In a mouse model of myocardial hypertrophy induced by transverse aortic constriction, HHP-EXO administration notably improved cardiac function, evidenced by better left ventricular ejection fraction and reduced hypertrophy.^[191]^ The therapeutic effect of HHP-EXO was primarily due to the suppression of hypertrophic signal molecules and the pivotal role of miRNA-148a, which inhibits the GP130 signaling pathway.^[191]^ This approach highlights the potential of using targeted exosomes as a novel, effective therapeutic strategy for cardiac hypertrophy, leveraging miRNA-mediated molecular pathways.^[191]^ Moreover, Vaskova and colleagues’ research on cardiac hypertrophy revealed that treatments with sacubitril/valsartan and valsartan influenced exosome production in human-induced pluripotent stem cell-derived cardiomyocytes.^[146]^ Significantly, they discovered that sacubitril/valsartan treatment led to a decrease in rno-miR-181a expression in these exosomes.^[146]^ This downregulation of miR-181a was linked to improved cardiac function in rodent models, evidenced by reduced myocardial fibrosis and hypertrophy.^[146]^ Their findings suggest that manipulating exosomal miR-181a levels through specific treatments can be a viable strategy for treating cardiac hypertrophy.^[146]^

Building on exosomal miRNA’s role in cardiac hypertrophy, Chen et al investigated the effects of exosomes derived from bone marrow mesenchymal stem cells (BM-MSCs) on cardiac hypertrophy and remodeling.^[184]^ Utilizing a transverse aortic constriction mouse model, they demonstrated that these exosomes significantly protected the myocardium from hypertrophy, reduced myocardial apoptosis and fibrosis, and preserved heart function under pressure overload.^[184]^ In vitro, the exosomes prevented hypertrophy in cultured myocytes stimulated with angiotensin II and promoted premature senescence in myofibroblasts, indicating an anti-fibrosis effect.^[184]^ A pivotal aspect of their findings was the role of miR-29a within the BM-MSCs exosomes.^[184]^ MiR-29a, a multifunctional component, was identified as a key regulatory cargo contributing to the cardiac protective effects, particularly in conditions of pressure overload.^[184,192]^ Being part of the miR-29 family, which is known to inhibit mRNAs involved in collagen and extracellular matrix production, miR-29a plays a significant role in mitigating cardiac hypertrophy.^[184]^ Their study suggested that BM-MSCs-derived exosomes, particularly through the action of miR-29a, offer a promising approach for treating heart failure associated with cardiac hypertrophy.^[184]^ Similarly, Wang et al^[193]^ highlighted the crucial role of miR-10a in reducing cardiac hypertrophy. They discovered that miR-10a, which is typically down-regulated in hypertrophic conditions, targets TBX5, a key factor in hypertrophy.^[193]^ Through experiments on a TAAC-induced rat model and angiotensin II-stimulated cardiomyocytes, they demonstrated that overexpressing miR-10a effectively diminishes hypertrophy markers and cell size, positioning it as a promising therapeutic target in exosome-based treatments for cardiac hypertrophy.^[193]^

Further extending this line of research, Zhao et al investigated exosomes from human-induced pluripotent stem cell-derived cardiomyocytes (hiPSC-CMs), particularly those with overexpressed cyclin D2 (CCND2OECMs), for their efficacy in cardiac hypertrophy treatment.^[194]^ Termed CCND2OEExos, these exosomes notably enhanced proliferation and hypoxia tolerance in cardiomyocytes.^[194]^ They facilitated an increase in proliferation markers like Ki67, PH3, and Aurora B and were effective in reducing apoptosis under hypoxic conditions.^[194]^ Central to their findings was the abundance of miR-302b-3p and miR-373-3p in CCND2OEExos, which are believed to augment cardiomyocyte proliferation, possibly by modulating the Hippo signaling pathway.^[194]^ This study positions CCND2OEExos, with their unique miRNA composition, as a potential therapeutic avenue for cardiac hypertrophy, focusing on bolstering cardiomyocyte proliferation and survival.^[194]^

### 6.2. Treating with Exosomal circRNAs

In addition to microRNAs, the therapeutic potential of exosomal circular RNAs in mitigating cardiac hypertrophy has been recognized in recent research. Zuo et al conducted a study focusing on the impact of a novel circular RNA, circ_0018553, present in exosomes from endothelial progenitor cells (EPCs), on cardiac hypertrophy induced by angiotensin II (Ang II).^[195]^ Their findings showed that circ_0018553 played a dual role: its silencing exacerbated cardiac hypertrophy, while its overexpression significantly reduced hypertrophy in cardiomyocytes.^[195]^ This circular RNA acted as a sponge for miR-4731, which targets the 3’ untranslated region (UTR) of sirtuin 2 (SIRT2), a key player in cardiac hypertrophy.^[195]^ The study highlighted that regulating the levels of miR-4731, either through overexpression or inhibition, had a direct impact on cardiac hypertrophy.^[195]^ This underscores the protective effect of EPC-derived exosomal circ_0018553 against Ang II-induced cardiac hypertrophy by influencing the miR-4731/SIRT2 signaling pathway.^[195]^

These findings highlight the potential of exosomal cargos, such as miRNAs and circRNAs, as innovative therapeutic agents for cardiac hypertrophy, offering new directions in heart disease treatment.

## 7. Conclusion and future perspectives

This review has comprehensively explored the multifaceted roles of exosomes in the mechanisms and therapeutic approaches of pathological cardiomyocyte hypertrophy. We delved into the intricate biogenesis of exosomes and their function as critical mediators in intercellular communication, impacting various facets of cardiac hypertrophy. Our examination extended from the molecular intricacies of cardiomyocyte hypertrophy linked to exosomes, to their potential as biomarkers and their significant implications in targeted therapy. Through this review, we underscored the pivotal role of exosomes in the pathophysiology and management of cardiac hypertrophy, offering insights into their utility in diagnostics and as innovative therapeutic vectors.

Although existing studies have detailed the mechanisms behind pathological cardiomyocyte hypertrophy and explored the role of exosomes, there’s still a need for more precise and thorough scientific research. Our article provides an initial summary of these findings, highlighting the necessity for further studies to conclusively prove these concepts. We aim to lay the groundwork for future research to deepen our understanding and fully exploit exosomes’ therapeutic potential in treating cardiac hypertrophy, emphasizing the importance of precision and rigorous scientific inquiry in advancing this field.

Building on the imperative for more rigorous scientific validation of exosomes’ roles, future research must also explore the nuanced interactions between exosomal components and their specific targets in the cardiac environment. This expanded focus will not only deepen our understanding of exosomal functions but also open avenues for novel therapeutic strategies aimed at mitigating cardiac hypertrophy. Given the demonstrated specificity and functionality of exosomes, there lies a critical opportunity to leverage these vesicles as vehicles for targeted drug delivery. This approach could exploit their innate ability to home in on specific cell types, thereby modulating key cellular functions in a targeted manner. Utilizing advanced gene editing technologies, such as CRISPR/Cas systems, could provide profound insights into the manipulation of exosomal content, enhancing the effectiveness of therapeutic interventions. Moreover, the development of standardized, reproducible protocols for exosome isolation, characterization, and administration will be paramount in bridging the gap between laboratory findings and clinical applications. Ultimately, the exploration of exosomes in cardiac hypertrophy holds the potential not only for groundbreaking therapeutic interventions but also for redefining our understanding of cellular communication in cardiac pathophysiology.

## Acknowledgments

We would like to take this opportunity to express our sincere gratitude to West China Hospital, Sichuan University, for their strong support of this research. Figure 1 was created using BioRender.com, and Figure 2 was created by Figdraw (accessed on 25 Nov 2023).

## Author contributions

**Methodology:** Fang Xie.

**Supervision:** Fang Xie.

**Validation:** Fengmei Zhang.

**Writing – original draft:** Lijun Zhang.

**Writing – review & editing:** Lijun Zhang, Beiyao Lu.
